# Sensitization of catastrophic cognition in cognitive-behavioral therapy for panic disorder

**DOI:** 10.1186/1471-244X-7-70

**Published:** 2007-12-10

**Authors:** Yumiko Noda, Yumi Nakano, Kiyoe Lee, Sei Ogawa, Yoshihiro Kinoshita, Tadashi Funayama, Norio Watanabe, Junwen Chen, Yuka Noguchi, Miyako Kataoka, Masako Suzuki, Toshi A Furukawa

**Affiliations:** 1 Department of Psychiatry and Cognitive-Behavioral Medicine, Nagoya City University Graduate School of Medical Sciences, Mizuho-cho, Mizuho-ku, Nagoya 467-8601, Japan; 2 Minami-Seikyo Hospital, Nagoya, Japan; 3 Department of Psychiatry, University of Southampton, Southampton, UK; 4 Faculty of Human Relations, Tokai Gakuin University, Gifu, Japan

## Abstract

**Background:**

Cognitive model of panic disorder have proposed that panic attacks result from the catastrophic misinterpretation of certain bodily sensations. Cognitive-Behavioral Therapy (CBT) for panic disorder aims to change these catastrophic cognitions. CBT intervention successfully caused reduction of catastrophic cognitions and symptomatic improvement in the majority of cases. However there are some patients who fail to modify their catastrophic cognitions or rather experience an increase in them during CBT treatment. It is clinically and theoretically important to understand about cognitive sensitization of panic disorder during CBT sessions. The purpose of the present study is 1) to clarify the baseline characteristics of panic patients who would experience sensitization of their catastrophic cognitions through the CBT treatment, and 2) to examine the course of symptomatic changes for them.

**Methods:**

Of ninety-five outpatients with panic disorder started the group CBT program for treatment of panic disorder, seventy-nine completer were classified as "cognitively sensitized (CS)" or "cognitive responding (CR)" or "no-responder" according to the difference of the Agoraphobic Cognitions Questionnaire score across treatment. We compared the CS and CR patients in terms of their baseline clinical characteristics. Then we assessed the symptomatic and functional changes for both groups.

**Results:**

At the start of the CBT program, despite of the same degree of panic disorder severity, CS scored significantly lower on ACQ score than CR. CS also showed significantly lower score on anticipatory anxiety compared to CR. At the end of treatment CS showed significant improvement in severity of panic disorder, although the degree of improvement was smaller than that for CR. Then CS would progressively reduce their agoraphobic fear and avoidance, and would improve their functional impairment up to three month of follow-up.

**Conclusion:**

Panic patients who would experience sensitization of their catastrophic cognitions through the CBT treatment could nonetheless gradually improve. They showed a relatively low level of catastrophic cognition and anticipatory anxiety before starting the CBT program. We might conclude that temporary sensitization of catastrophic cognition may be necessary before improvement especially among those with initially low catastrophic body sensation fears and that we need not be concerned too much with temporary increase in catastrophic cognition in the process of CBT for panic disorder.

## Background

A number of studies have supported the effectiveness of cognitive behavioral therapy (CBT) for panic disorder [1-3]. Several studies also suggested that CBT is effective treatment for panic patients who have failed to respond to adequate pharmacotherapy [4-7].

Cognitive model of panic disorder[8] have proposed that panic attacks result from the catastrophic misinterpretation of certain bodily sensations. Patients have the tendency to perceive essentially benign and normal sensations as evidence of imminent danger. For example, palpitations are misinterpreted as a signal of a heart attack, dizziness as evidence impeding loss of control, etc. Goldstein and Chambless [9] labelled this fear of experiencing anxiety or panic attacks as "fear of fear". This leads to hypervigilance about bodily sensations, increased arousal of the sympathetic nervous system, more physical sensations, and heightened anxiety, which spiral into a panic attack.

CBT for panic disorder aims to change these catastrophic cognitions[10]. Taylor [11] reported that modifying patients' catastrophic misinterpretations of bodily sensations results in significant reductions in panic. Although it is unclear whether change in cognition instigates symptomatic change or not, many studies suggest that successful treatment was associated with a reduction in catastrophic cognitions [10,12-14]. These studies reported that CBT intervention successfully caused reduction of catastrophic cognitions and symptomatic improvement in the majority of cases. However there are some patients who fail to modify their catastrophic cognitions or rather experience increase in them during CBT treatment. In a biological challenge study for panic disorder patients, Beck and Shipherd[15] found that there were two distinct response patterns to repeated presentation of physical sensations: habituation of fear and fear sensitization. It is clinically and theoretically important to understand about cognitive sensitization of panic disorder during CBT sessions. However the baseline characteristics of the patient who would sensitize their catastrophic cognition during CBT, and the course of panic disorder for them are not well known.

The purpose of present study is 1) to clarify the baseline characteristics of panic patient who would experience sensitization of their catastrophic cognitions through the CBT treatment, and 2) to examine the course of symptomatic changes for them.

## Methods

### Subjects

Subjects were 137 consecutive patients who sought treatment for panic disorder at Nagoya City University Hospital Department of Psychiatry between June 2001 and March 2006. Of 137 patients, 95 subjects attended the group cognitive-behavioral therapy program.

At the start of the CBT program, all the patients met the following entry criteria:(a) principal Axis I diagnosis of panic disorder with or without agoraphobia according to the DSM-IV(Diagnostic and Statistical Manual of Mental Disorders, Fourth Edition) criteria, as assessed by the Structured Clinical Interview for DSM-IV(SCID) [16]; (b) absence of a history of psychosis and current substance-use disorder; (c) highly motivated to undergo CBT. Patients with other comorbid anxiety disorders and/or major depressive disorders were admitted to the CBT program after the symptoms of these disorders had abated and they were able to participate in the program. At the start of the CBT program, of ninety-five patients, there are five with social phobia, 3 with obsessive-compulsive disorder, 3 with specific phobia, 5 with post-traumatic stress disorder and one with generalized anxiety disorder. As for depression at the start of the CBT program, there are three patients with present major depressive episode and five with dysthymic disorder.

Use of antidepressants to control their anxiety and/or depression was permitted throughout the CBT period. Because these drugs do not interfere with CBT treatments [17]. In case the patients were using benzodiazepines regularly or occasionally during the daytime to control their anxiety, we advised them to taper and stop usage or to switch to an antidepressant before starting the CBT program, since these drugs may interact negatively with exposure treatment [18]. Some patients reported that they had not been able to stop taking anxiolytic benzodiazepine by the start of the CBT program. Medications for symptoms other than anxiety were not restricted, so patients were allowed to use short-acting benzodiazepine hypnotic.

At the start of the CBT program, 56 patients used antidepressants, 19 patients used benzodiazepines regularly or occasionally.

The Ethics Committee of Nagoya City University Graduate School of Medical Sciences approved the study protocol and all subjects provided written informed consent after full explanation of the purposes and procedures of the study.

### Treatment

CBT program used in this study was based on the CBT program developed by the Clinical Research Unit for Anxiety and Depression at the University of New South Wales, Sydney, Australia [19]. This treatment consists of five major components: (a) psycho-education concerning the nature, causes and maintenance of anxiety and panic; (b) breathing retraining; (c) cognitive restructuring; (d) graded situational exposure to reduce agoraphobic avoidance; (e) interoceptive exposure to reduce patients' fear of somatic sensations. Patients were assigned homework after each session.

One group consisted of three to four patients. Two trained therapists (psychiatrists or clinical psychologists with at least 2 years of clinical experience) conducted two-hour highly structured sessions once a week for ten weeks, using a detailed manual.

The senior psychiatrist, who had more than 15 years' experience as a clinician and had observed the PD-CBT program at St. Vincent's Hospital, trained the other therapists. With the aim of training, the junior psychiatrists or psychologists attended a series of CBT sessions as co-therapist, which facilitated mainly by the senior therapist. Then trained junior psychiatrist or psychologist began to lead a group CBT sessions assisted by another junior therapist. The senior psychiatrist supervised the other therapists at a CBT clinical conference held once a week.

### Measures

Before enrolment into the CBT program, all participants received a semi-structured interview judging the anxiety and mood disorders section of the SCID and the Panic Disorder Severity Scale (PDSS). This interview was conducted by one of the clinicians carrying out the CBT program. Then at the start of the program, all patients completed five self-report questionnaires. To assess the intensity of catastrophic cognitions, patients were asked to fill out the Agoraphobic Cognitions Questionnaire (ACQ). To evaluate the clinical status and personality characteristics, patients completed the Work, Home and Leisure Activity Scales (WHLS), the Fear Questionnaire-agoraphobic subscale (FQ-ag), the Mobility Inventory for agoraphobia-alone subscale (MI-alone), and the NEO-Five Factor Inventory (NEO-FFI). The same sets of instruments except for the personality inventory were repeated at the end of the program. Three months after the end of the program, we mailed follow-up questionnaires including ACQ, FQ-ag, MI-alone, and WHLS. Details of these instruments are described below. Since we followed up by post mail, PDSS which is based on a semi-structured interview was not administered at 3-month follow-up.

#### Agoraphobic Cognitions Questionnaire (ACQ)

The ACQ is a 14-item self-report instrument to assess "fear of fear" or cognitions concerning catastrophic consequences of experiencing anxiety. Each item is rated on a five-point scale ranging from 1(thought never occurs) to 5(thought always occurs), according to the frequency with which this thought occurred when the client was anxious. Good reliability and validity have been shown for both the original and the Japanese versions of this questionnaire[20,21].

#### Panic Disorder Severity Scale (PDSS)

The PDSS is a seven-item interview-based scale of panic disorder severity in which the clinician rates the severity of seven features of panic disorder on a scale rating from 0 (none) to 4 (extreme). The features that are rated include frequency of panic attack, distress during panic attack, anticipatory anxiety, agoraphobic fear/avoidance, interoceptive fear/avoidance, work impairment/distress, and social impairment/distress. Adequate inter-rater reliability and validity have been reported for both the original and the Japanese versions [22,23].

#### The Work, Home and Leisure Activities Scale (WHLS)

The Work, Home and Leisure Activity Scales was designed to assess role functioning in the areas of work, home management, social leisure, and private leisure activities. Each item is rated on a nine-point scale ranging from 0 (not at all impaired) to 8 (very severely impaired). Satisfactory reliability and construct validity have been reported [24].

#### Fear Questionnaire-agoraphobia subscale (FQ-Ag)

The Fear Questionnaire-agoraphobia subscale is a self-report instrument for measuring the severity of agoraphobic avoidance of five typical situations for agoraphobia. Each situation is rated on a nine-point scale ranging from 0 (would not avoid it) to 8 (always avoid it) to show how much he/she would avoid each of the listed situations because of fear or other unpleasant feelings. Test-retest reliability and factor validity have been confirmed [25].

#### Mobility Inventory for agoraphobia-alone subscale (MI-alone)

The Mobility Inventory for agoraphobia is a self-report instrument for measuring the severity of agoraphobic avoidance. Patients are asked to rate on a five-point scale ranging from 1(never avoid) to 5(always avoid) to show how they feel about 31 places or situations that they may avoid because of anxiety or phobia, when they are alone. This instrument has been shown good reliability and construct validity [26,27].

#### NEO-Five Factor Inventory (NEO-FFI)

The NEO-FFI is a 60-item self-reported inventory aimed to assess five personality factors of neuroticism, extraversion, conscientiousness, openness, and agreeableness [28].

### Statistical analyses

To estimate the change of catastrophic cognition, we calculated the differences in ACQ total score across the treatment for each subject. Then we considered the standard error of measurement (http://www.w3.org/1998/Math/MathML1471-244X-7-70-i1SEM=σ1−γMathType-MTEF MathType@MTEF@5@5@+=feaafiart1ev1aaatCvAUfKttLearuWrP9MDH5MBPbIqV92AaeXatLxBI9gBaebbnrfifHhDYfgasaacPC6xNi=xH8viVGI8Gi=hEeeu0xXdbba9frFj0xb9qqpG0dXdb9aspeI8k8fiI+fsY=rqGqVepae9pg0db9vqaiVgFr0xfr=xfr=xc9adbaqaaeGacaGaaiaabeqaaeqabiWaaaGcbaGaee4uam1aaSbaaSqaaiabbweafjabb2eanbqabaGccqGH9aqpiiGacqWFdpWCdaGcaaqaaiabigdaXiabgkHiTiab=n7aNbWcbeaaaaa@35D2@: *σ* = standard deviation of normal population, *γ* = test-retest reliability coefficient). Since there was no available data revealing the stability of ACQ for a Japanese sample, we calculated the S_EM _for ACQ by using the data from Chambless *et al.*[20], and Chambless[29]. Based on the calculated S_EM _for ACQ total score (S_EM _= 2.41), we set the cut-off point at 3 as minimum change score. Each subject was classified as a "cognitive responding (CR)," or a "cognitively sensitized (CS)" based on their differences of ACQ total score across the treatment. The subject whose ACQ score at post-treatment was 3-point or more lower than pre-treatment ACQ score was classified as CR, and those whose post-treatment ACQ score was 3-point or more higher than pre-treatment ACQ score was classified as CS. Others who change the ACQ ranged from +2 to -2 across the treatment were considered as non-responder in cognition.

Following categorization of subjects, we compared the demographic data and the personality traits at the start of the CBT program among groups, using analysis of variance (ANOVA) for continuous variables and chi-squared test of independence for categorical variables. If the data distribution did not follow the normality assumption, Kruskal-Wallis Test was utilized.

We then focused on the differences between the CS and CR patients. First we compared these two groups in terms of their baseline clinical characteristics. The group mean values were compared using the independent Student *t*-test, as long as the variables were normally distributed within each group and the variation of scores in the two groups were not reliably different. If the data distribution did not follow the normality assumption, Mann-Whitney U test was utilized.

For the assessment of changes in symptom severity and functional impairment across treatment in both groups, Student's t-test for dependent samples was used. If the data distribution did not follow the normality assumption, Wilcoxon rank-sum test was utilized. The within-group treatment effect size (ES) was calculated by Cohen's *d*.

Further ANOVA for repeated measures with PDSS total score as the dependant variable was performed to evaluate whether the degree of improvement differed between the two groups.

To examine the course of symptomatic changes for the two groups, we conducted a 2(group: CS vs. CR) × 3(time: baseline vs. post-treatment vs. three-month follow-up) repeated measures ANOVA with FQ-ag score, MI-alone score and WHLS as the dependant variable, followed by the post-hoc Dunnett's test when appropriate. All data undergoing ANOVA were tested for assumption of sphericity with Mauchly's test. In cases the sphericity did not hold, we adjusted the degree of freedom for the F-test using the Greenhouse-Geisser epsilon.

Statistical analyses were performed using the SPSS program version 15.0. All the statistical tests were two-tailed, and results were considered significant when p < 0.05.

## Results

Of ninety-five patients who started the CBT program, sixteen patients withdrew from the program before its completion, because of symptom improvement, lack of improvement, inconvenience of attending the program, concurrent physical illness or worsening of depression. The seventy-nine completers (26 men and 53 women) with a mean age of 36.1(SD = 11.3) were classified as CS(N = 11) or CR(N = 52) or no-responder(N = 16) according to the difference of ACQ score across the treatment. Table 1 presents baseline sample demographic and personality characteristics. At the start of the CBT program, there were no significant differences between the four groups.

**Table 1:** Baseline demographic and personality characteristics of the subjects

	Cognitively sensitized (N = 11)	Cognitive responding (N = 52)	No-response in cognition (N = 16)	Drop-out from treatment (N = 16)
Female	6(54.5%)	34 (65.4%)	13(81.3%)	12(75.0%)
With agoraphobia	10(90.9%)	49(94.2%)	14(87.5%)	15(93.8%)
Mean age (SD)^a^	37.5(13.3)	35.0(9.7)	38.9(14.4)	34.5(11.4)
Mean age at onset of PD (SD)^a^	28.3(13.8)	29.8(9.7)	39.9(12.8)	29.1(9.4)
Mean yeas of duration of panic disorder (SD)^a^	9.2(12.1)	5.2(5.1)	9.1(11.7)	5.4(6.2)
Severity of panic disorder: PDSS^b ^total score at baseline (SD)^a^	11.1(4.1)	13.2(4.7)	9.3(4.5)	12.5(4.9)
Medication at start of CBT session				
Antidepressants	9(81.8%)	31(59.6%)	9(56.3%)	7(43.8%)
Benzodiazepines	5(45.5%)	9(17.3%)	3(18.8%)	2(12.5%)
NEO-FFI^c^(SD)				
Neuroticism	22.6(11.8)	28.1 (7.6)	22.3(9.4)	27.5(8.7)
Extraversion	25.6(11.0)	25.6(6.7)	28.3(9.6)	26.2(10.3)
Openness	25.2(9.4)	27.6(5.9)	26.1(5.6)	30.4(5.8)
Agreeableness	31.6(10.6)	32.1(7.2)	36.7(6.1)	30.4(5.1)
Conscientiousness	24.8(12.4)	28.6(6.7)	27.5(8.0)	28.3(9.3)

No significant differences for all comparisons.

a:SD = Standard Deviation b:PDSS = Panic Disorder Severity Scale

c:NEO-FFI = NEO-Five Factor Inventory

Table 2 shows the clinical characteristics for CS and CR patients at baseline. CS patients scored significantly lower on the ACQ than CR patients. The mean score of PDSS (11.1 for CS, 13.2 for CR) indicated a high-to-moderate level of panic disorder severity for both groups and the total scores were not significantly different at baseline between the two groups. On the item level, frequency of panic attack, distress of attack, agoraphobic fear/avoidance, and impairment of work or social functioning did not differ significantly. However, CS patients presented a significantly lower score than CR patients on anticipatory anxiety and non-significant trend to a lower score on interoceptive fear/avoidance. The scores of FQ-ag, MI-alone and WHLS did not differ between the groups.

**Table 2:** Clinical status at baseline by group

	Group		p*
		
	CS^a^(n = 11) Mean(SD)	CR^b^(n = 52) Mean(SD)	
ACQ^c^	22.9(4.7)	30.8(8.8)	.003
PDSS^d ^total score	11.1(4.1)	13.2(4.7)	.160
frequency of panic	1.1(0.5)	1.3(0.9)	.574
distress duringpanic	2.4(1.1)	2.5(1.3)	.655
anticipatory anxiety	1.2(1.1)	1.9(1.0)	.041
agoraphobic fear/avoidance	2.0(1.0)	2.2(1.1)	.548
interoceptive fear/avoidance	0.6(0.8)	1.2(1.1)	.078
impairment of work functioning	1.82(1.2)	2.1(1.1)	.572
impairment of social functioning	2.0(1.0)	2.1(1.1)	.785
FQ-ag^e^	13.6(12.2)	13.2(9.8)	.971
MI-alone^f^	2.4(0.9)	2.6(1.0)	.615
WHLS^g^	11.3(8.3)	10.9(5.4)	.894

*:Student's t-test for independent samples or Mann-Whitney U test

a: CS = Cognitively sensitised b: CR = Cognitive responding

c:ACQ = Agoraphobic Cognitions Questionnaire, d:PDSS = Panic Disorder Severity Scale, e:FQ-ag = Fear Questionnaire-agoraphobia subscale, f:MI-alone = Mobility Inventory for agoraphobia-alone subscale, g:WHLS = The Work, Home and Leisure Activities Scale

By definition, as can be seen in Table 3, ACQ scores increased significantly across treatment among CS patients. The mean score of ACQ moved from 22.9(SD = 4.7, range: 18 to 33) at baseline to 27.7(SD = 5.0, range: 21 to 36) at endpoint. For CR patients, ACQ score decreased significantly. Their group mean score moved from 30.8(SD = 8.8, range: 18 to 56) at baseline to 20.7(SD = 6.1, range: 14 to 42) at endpoint.

For both groups, PDSS total scores improved significantly; CS patients' mean score moved from 11.1(SD = 4.1) at baseline to 9.1(SD = 4.4) at endpoint. CR patients' mean score moved from 13.2(SD = 4.7) at baseline to 5.9(SD = 4.3) at endpoint. Repeated measures ANOVA for PDSS total score showed a significant main effect of time (F(1,60) = 32.239, p < 0.001), and Group × Time interaction (F(1,60) = 10.80, p = 0.002). The degree of improvement differed between two groups.

**Table 3:** Changes of panic symptoms across the treatment, including effect sizes

	Cognitive I y Sensitized patients (N = 11)				Cognitive Responding patients (N = 52)			
	
	Baseline	Post-treatment	p^a^	Effect size^b^	Baseline	Post-treatment	p^a^	Effect size^b^
ACQ^c^	22.9(4.7)	27.7(5.0)	.<000	-2.34	30.8(8.8)	20.7(6.1)	<0.00	1.59
PDSS^d^-total	11.1(4.1)	9.1(4.4)	.047	0.62	13.2(4.7)	5.9(4.3)	<0.00	1.41
PDSS^d^-item							<0.00	
frequency of panic	1.1(0.5)	0.9(0.5)	.317	0.30	1.3(0.9)	0.8(0.8)	<0.00	0.56
distress duringpanic	2.4(1.1)	2.4(1.1)	.732	0.03	2.5(1.4)	1.4(1.1)	<0.00	0.70
anticipatory anxiety	1.2(1.1)	1.1(0.6)	.792	0.08	1.9(1.0)	0.8(0.8)	<0.00	1.01
agoraphobic fear/avoidance	2.0(1.0)	1.5(0.8)	.107	0.53	2.2(1.1)	0.9(0.9)	<0.00	1.08
interoceptive fear/avoidance	0.6(0.8)	0.7(0.9)	.603	-0.15	1.2(1.1)	0.5(0.8)	<0.00	0.60
impairment of work functioning	1.8(1.2)	1.5(1.3)	.206	0.39	2.1(1.2)	0.7(1.2)	<0.00	1.03
impairment of social functioning	2.0(1.0)	1.1(1.1)	.039	0.74	2.1(1.1)	0.9(1.0)	<0.00	1.08
FQ-ag^e^	13.6(12.2)	10.6(8.2)	.153	0.42	13.2(9.8)	5.1(6.4)	<0.00	0.79
MI-alone^f^	2.4(0.9)	2.1(0.7)	.070	0.61	2.6(1.0)	1.7(0.7)	<0.00	1.59
WHLS ^g^	3.2(2.1)	2.4(1.8)	.026	0.78	2.7(1.4)	1.3(1.3)	<0.00	0.53

a: Student's t-test for dependent samples or Wilcoxon rank-sumtest, b: Within-group effect size (ES) by Cohen's *d*. c:ACQ = Agoraphobic Cognitions Questionnaire, d:PDSS = Panic Disorder Severity Scale, e:FQ-ag = Fear Questionnaire-agoraphobia subscale, f:MI-alone = Mobility Inventory for agoraphobia-alone subscale, g:WHLS = The Work, Home and Leisure activities Scale

With regard to symptomatic and functional changes across treatment, CR patients showed significant improvement for all variables. In contrast, CS patients significantly improved in impairment of social functioning and WHLS, and showed non-significant trend to improve in MI-alone.

Results of repeated measures ANOVA which examined the courses of symptomatic changes from baseline to three month follow-up for the two groups are presented in Table 4. For all variables, we found a significant main effect of time and no significant Group × Time interaction.

**Table 4:** Results from repeated measure ANOVA for Change of panic symptoms

	p-value result from repeated measure ANOVA		
Dependant variable	Time	Group	Interaction

FQ-a^a^	<0.00	0.44	0.39
MI-alone^b^	<0.00	0.93	0.11
WHLS^c^	<0.00	0.04	0.38

a:FQ-ag = Fear Questionnaire-agoraphobia subscale, b:MI-alone = Mobility Inventory for agoraphobia-alone subscale, c:WHLS = The Work, Home and Leisure activities Scale

As can be seen in Table 5, post hoc Dunnett tests revealed that post-treatment scores of CR patients were significantly reduced compared to baseline scores for all measures. And 3-month follow-up scores of CR patients were also significantly reduced compared to baseline scores. In contrast, at the end of the program CS patients showed non-significant trend to reduce their post-treatment scores in MI-alone and WHLS, and did not show significant reduction in FQ-ag. Whereas compared to baseline scores, 3-month follow-up scores of CS patients were significantly reduced for FQ-ag and MI-alone, and showed non-significant trend to be reduced for WHLS.

**Table 5:** Changes of panic symptoms at endpoint and 3-month follow-up

Dependant variable	Time	CS^a^		CR^b^	
		
		mean(SD)	p-value*	mean(SD)	p-value*
FQ-ag^c^	baseline	13.6(12.2)		13.2(9.8)	
	end-point	10.6(8.2)	0.33	5.1(6.4)	<0.00
	3 month-f/u	7.3(7.7)	0.04	4.7(6.3)	<0.00
MI-alone^d^	baseline	2.4(0.9)		2.6(1.0)	
	end-point	2.1(0.7)	0.09	1.7(0.7)	<0.00
	3 month-f/u	1.8(0.8)	0.001	1.8(0.9)	<0.00
WHLS^e^	baseline	3.2(2.1)		2.7(1.4)	
	end-point	2.4(1.8)	0.05	1.3(1.3)	<0.00
	3 month-f/u	2.4(1.8)	0.09	1.3(1.5)	<0.00

*:Result from post hoc test using Dunnett' method

a: CS = Cognitively sensitised b: CR = Cognitive responding c:FQ-ag = Fear Questionnaire-agoraphobia subscale, d:MI-alone = Mobility Inventory for agoraphobia-alone subscale, e:WHLS = The Work, Home and Leisure activities Scale

## Discussion

With regard to the first objective of the present study, we were able to delineate the baseline characteristics of panic patient who would experience sensitization of their catastrophic cognitions through the CBT treatment as follows. Despite the same degree of panic disorder severity, CS patients showed relatively low level of catastrophic cognitions than CR patients preceding CBT treatment. Furthermore, CS patients reported lower level of anticipatory anxiety and interoceptive fear/avoidance before treatment. These findings are generally consistent with prior research conducted by Beck & Shipherd[15] who found that panic disorder patients who showed fear sensitization during repeated CO2 inhalation sessions had shown a relatively low level of anticipatory anxiety preceding CO2 inhalation.

The second purpose of this study was to examine the course of symptomatic change of CS patients. We found that at the end of treatment CS patients showed significant improvement in severity of panic disorder, although the degree of improvement was smaller than that for CR patients. The improvement for CS patients at the end of treatment was mainly due to restoration of social functioning. This improvement for CS patients appears to continue at least for three months following the end of treatment. Consistent with previous findings [3,11,14,30], CR patients were significantly and clinically improved at the end of CBT program and they would continue to improve up to three month follow-up. In contrast, CS patients showed lower improvement compared to CR patients at the end of program, but then they would progressively reduce their agoraphobic fear and avoidance, and would improve their functional impairment up to three month of follow-up.

It is interesting to note that panic patients who would experience sensitization of their catastrophic cognitions through the CBT treatment could nonetheless gradually improve. It has already been pointed out that exposure to feared situations or bodily sensations can initially increase anxiety. Otto et al. emphasized that these experience help patients to learn that they can be "OK" despite the presence of anxiety [3]. We may then be able to postulate that CS patients had been taking the strategy of cognitive avoidance of the threat cues before attending CBT treatment. Koster et al. reported the paradoxical effects of suppressing anxious thoughts [31]. They found that during thought suppression, self-reported anxiety and frequency of anxious thoughts did not increase, and duration of anxious thoughts decreased. After thought suppression, participants experienced an increase in self-reported anxiety and the frequency of anxious thoughts. Thus for anxious patients the strategy of cognitive avoidance might play a role in maintaining anxiety disorder. When cognitive avoidance is present, then sensitization of catastrophic cognitions might be clinically meaningful in CBT for panic disorder.

Several limitations of current study should be mentioned. First, the procedure of categorization that classified patients into "cognitively sensitized" or "cognitive responding" may appear rather arbitrary. A different categorization strategy may have been possible but in the absence of agreed-upon procedure, we set the S_EM _as the cut-off point. Second, the sample size was rather small to generalize our results and some of our negative findings might be due to lack of power. However, we can be sure of what positive findings we were able to identify. Third, the study did not include a control group and one cannot be sure if the significant reduction in panic symptomatology observed in the present cohort might be due to passage of time rather than to CBT treatment per se. Fourth, we did not confirm the change of symptomatorogy for depression with objective measure during the treatment and follow-up phase. Therefore we could not perfectly exclude the possibility that the results of this study affected any symptom of depression. Fifth, we did not confirm whether the patient underwent other treatments in the post treatment follow-up phase. Therefore strictly we couldn't rule out the possibility the improvement of patients due to some other treatment.

Casey *et al.*[12] concluded in their study that it is necessary to include measures of positive cognitions (e.g. self efficacy in dealing with anxiety thought) in assessing the impact of catastrophic cognitions. We didn't address the positive cognitions in this study. Further research will be needed to investigate how self efficacy changes, especially in CS patient.

## Conclusion

In conclusion, we found that CS patients had a lower level of catastrophic cognition and anticipatory anxiety than CR patients at baseline before starting the CBT program.

Through and at three months after the CBT program, CR patients demonstrated robust and continued improvement in symptomatology and functioning, while CS patients lagged behind CR patients, because they scored worse at the end of acute phase treatment than CR patients but tended to catch up with them at three-month follow-up. When clinically paraphrased we might conclude that temporary sensitization of catastrophic cognition may be necessary before improvement especially among those with initially low catastrophic body sensation fears and that we need not be concerned too much with temporary increase in catastrophic cognition in the process of CBT for panic disorder.

## Competing interests

TAF has received research funds and speaking fees from Asahi Kasei, Astellas, Dai-Nippon, Eisai, Eli Lilly, GlaxoSmithKline, Janssen, Kyowa Hakko, Meiji, Organon, Pfizer, Tsumura, Yoshitomi, and Zelia. All the other authors declare that they have no competing interests to declare.

## Authors' contributions

YNoda was the primary investigator; YNakano, KL, SO, YK, TF, NW, JC, YNoguchi, MK, MS and TAF performed the clinical investigation (diagnosis, treatment, assessment and follow-up phases); TAF participated on the design of the study and supervised the overall conduct of the study. All authors approved the final manuscript.

## Pre-publication history

The pre-publication history for this paper can be accessed here:


